# Effects of Medium Composition and Genetic Background on *Agrobacterium*-Mediated Transformation Efficiency of *Lentinula edodes*

**DOI:** 10.3390/genes10060467

**Published:** 2019-06-19

**Authors:** Lianlian Yan, Ruiping Xu, Yan Zhou, Yuhua Gong, Shenghong Dai, Haiyang Liu, Yinbing Bian

**Affiliations:** 1 Institute of Applied Mycology, College of Plant Science and Technology, Huazhong Agricultural University, Wuhan 430070, China; yylaputa@163.com (L.Y.); mushroomhzau@163.com (R.X.); yanzhoufbw@mail.hzau.edu.cn (Y.Z.); gongyuhua@mail.hzau.edu.cn (Y.G.); Shongdai@163.com (S.D.); wwqbrad@163.com (H.L.); 2 Key Laboratory of Agro-Microbial Resource Comprehensive Utilization, Ministry of Agriculture, Huazhong Agricultural University, Wuhan 430070, China

**Keywords:** *Lentinula edodes*, medium composition, *Agrobacterium*-mediated transformation, genotype, transformation efficiency

## Abstract

The establishment of genetic transformation method is crucial for the functional genomics research in filamentous fungi. Although the transformation method has been developed in several types of fungi, a highly efficient and convenient transformation system is desperately needed in *Lentinula edodes*. Present work established the *Agrobacterium*-mediated transformation (ATMT) of basidiomycete *L. edodes* in both monokaryon and dikaryon mycelia by using constructed binary plasmid pCAMBIA-1300-GFP. Then, the transformation efficiency of ATMT was evaluated by using different mediums for recipient incubation and different varieties of *L. edodes*. The results showed that in dikaryon strain W1, the positive hygromycin-resistant transformants was observed in all medium with the positive frequency of selected transformants that ranged from 0 to 30%. While in the monokaryon strain W1-26, only the millet medium group obtained positive transformants with a positive frequency of 75.48%. Moreover, three dikaryotic wild strains (YS55, YS3334, and YS3357) and two dikaryotic cultivated strains (W1 and S606) showed the highest transformation efficiency, with 32.96% of the germination frequency, and 85.12% of positive frequency for hygromycin-resistant transformants. This work demonstrated that *Agrobacterium*-mediated transformation was successfully performed in *L. edodes*, and the genotype of recipients as well as the medium for mycelial incubation were suggested to play key roles in determining the transformation efficiency. These findings may provide new avenues for the genetic modification of edible mushroom and may extend the cognition of DNA-mediated transformation in filamentous fungi.

## 1. Introduction

Filamentous fungi encompass diverse groups and are widely distributed in nature. Basidiomycetes, an indispensable member in filamentous fungi, play important roles in agriculture, industry, and medicine [1]. Genome sequences of hundreds of filamentous fungi have been established (http://genome.jgi.doe.gov/). With the rapid development of fungal genomic sequencing, mushroom research has entered an era of functional studies. The genomes of several species have been sequenced and published, such as *Coprinosis cinerea*, *Schizophyllum commune*, *Agaricus bisporus*, *Ganoderma lucidum*, *Lentinula edodes*, etc. [2,3,4,5,6]. *L. edodes*, also called Xianggu or shiitake, one of the edible and medicinal mushrooms, is also known as a white rot species with a strong ability to degrade lignin [7]. In China, it is widely cultured by sawdust-based cultivation. Transcriptome or proteome analyses have been used for obtaining differentially expressed genes (DEGs) [8,9]. For instance, various DEGs involved in heat stress and degradation of different carbon sources have been isolated [7,10]. However, only four *L. edodes* genes have been revealed about the functions by genetic transformation technologies [11,12,13,14]. Despite the publication of several articles on *L. edodes* transformation, the transformation efficiency of these methods is insufficient to support large-scale functional genomics research. 

Since the first DNA transformation was accomplished in *Neurospora crassa*, DNA-mediated transformation systems and subsequent gene-function assessment have been developed in an increasing number of filamentous fungi [15]. The transformation methods available for filamentous fungi include protoplast-mediated transformation (PMT), *Agrobacterium*-mediated transformation (ATMT), electroporation, and restriction enzyme-mediated integration (REMI) [16]. In 1998, *L. edodes* was transformed by REMI based on PMT transformation method using polyethylene glycol (PEG) [17]. This method was optimized two times, and the highest transformation efficiency was only 3.6 transformants per µg DNA per 10^7^ protoplasts [18,19]. In 2006, the PEG transformation method was used again to transform *L. edodes*, the results showed the transformation efficiencies were 24–28 times greater than previously reported, but their protocol relied on protoplasts as recipient tissue [20]. The low yield and low regeneration rate of it for some species have limited the wide application of the protoplast-based method [15]. In 2007, electroporation was applied to transform *L. edodes* using basidiospores or mycelial fragments as recipients [21]. Nevertheless, this method was not widely used, considering that electroporation is highly toxic to the cells and basidiospores are more difficult to distinguish the phenotypic variations caused by genetic differentiations or transformation mutants [22]. The *Agrobacterium*-mediated transformation system was applied successfully to transform *L. edodes* by using the mycelium of a monokaryon strain as a recipient, while it failed when using the mycelium of a dikaryon strain [23]. Genetic transformation systems were established in several fungi: The basidiomycete fungi *A. bisporus* and *G. lucidum*, the cucurbit powdery mildew pathogen *Podosphaera xanthii*, the ascomycete *Morchella importuna* [23,24,25,26]. Regardless of the feasibility of existing transformation systems, most fungi still have no genetic transformation systems available.

ATMT method has been shown to be more stable and efficient than conventional transformation methods [22]. It has been successfully used for the transformation of a variety of filamentous fungi, and it is accessible for research involved in gene silencing or overexpression [11,27]. Compared with the PEG-mediated transformation method, the ATMT method has many advantages, including diversified transformation recipients, generating stable transformants, high transformation efficiency, and generating knock-in mutations, due to the random insertion of T-DNA into the genome as a single copy [28]. Mushrooms, spores, mycelia, protoplasts, and tissues of fruiting bodies have been subjected to ATMT [29]. Because of the high regeneration efficiency and easy preparation, mycelia were used in this study. Successful ATMT of filamentous fungi relies on several factors, such as acetosyringone (AS) concentration, strains of *A. tumefaciens*, cultivation conditions, recipient tissue, and so on [30]. 

In plant, many archived studies showed that genotype of the recipient plants affects the efficiency of genetic transformation, while few similar researches were done in fungi [31]. Previously, the whole genomes of 39 wild and 21 cultivated strains of Chinese *L. edodes* were resequenced, two major groups were revealed by using inter-simple sequence repeat (ISSR) and sequence-related amplified polymorphism (SRAP) marker [32,33]. In another study, population structure analysis identified two unambiguous genetic groups among 88 wild strains of Chinese *L. edodes*, which correspond to two geographic regions from which the samples were collected [34]. Here, three wild and two cultivated strains with different genotypes were chosen as recipient to compare the transformation efficiency. Selection of a suitable strain for genetic transformation may be one of the key factors to improve transformation efficiency.

The ATMT transformation efficiency for *L. edodes* can be influenced by many factors. In this study, we tried to establish a general and relatively efficient ATMT method for transformation of several *L. edodes* strains. 

## 2. Materials and Methods

### 2.1. Strains and Culture Conditions

Dikaryotic wild strains (YS55, YS3334, and YS3357) and cultivated strains (W1 and S606) of *L. edodes* and a monokaryotic W1-26 strain derived from the W1 strain were used for genetic transformation [6,34,35]. Strains and transformants were maintained on potato dextrose agar medium (PDA). Two *A. tumefaciens* strains EHA105 and AGL1 were cultured in Luria-Bertani media (LB) at 28 °C. 

### 2.2. Plasmid Construction

**Figure 1:**
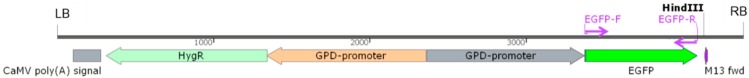
The T-DNA region of plasmid pCAMBIA-1300-GFP. *hygR*: the hygromycin B phosphotransferase gene as a selection marker; *EGFP*: the enhanced green fluorescent protein as a reporter gene; GPD-promoter: the glyceraldehyde-3-phosphate dehydrogenase promoter of *L. edodes*; LB: left border; RB: right border.

The promoter of *L. edodes* glyceraldehyde-3-phosphate dehydrogenase gene (*Legpd*), amplified from W1-26 by primers Pgpd-F and Pgpd-R, was employed to promote the expression of hygromycin-B-resistant gene (*hygR*) and reporter gene *eGFP*. The *eGFP* gene, encoding an enhanced green fluorescent protein, was amplified from pEGFP-1 by the primer pair gfp-F/gfp-R (Table 1). First, the *Legpd* promoter was introduced to drive *hygR* gene expression instead of the CaMV 35s promoter in pCAMBIA 1300 (CAMBIA, Canberra, Australia). The new vector was named pCAMBIA-1300-g. Then, the *Legpd* promoter and *eGFP* gene were cloned into pCAMBIA-1300-g to construct a binary vector pCAMBIA-1300-GFP using ClonExpress Multis One Step Cloning Kit (Vazyme, Nangjing, China) according to the manufacturer’s protocol (Figure 1). Finally, the pCAMBIA-1300-GFP vector was introduced into *Agrobacterium tumefaciens* strains EHA105 or AGL1 for further transformation experiments.

### 2.3. A. Tumefaciens-Mediated Transformation 

For transformation experiments, the single colonies of *A. tumefaciens* strains EHA105 and AGL1 containing pCAMBIA1300-GFP were grown at 28 °C for 24 h in 1 mL LB liquid medium containing 50 μg·mL^−1^ rifampicin and 50 μg·mL^−1^ kanamycin. Then, 1 mL bacterial suspension was added to 100 mL minimal medium (MM) [36] with 50 μg·mL^−1^ kanamycin, and grown at 28 °C for 2 days. Finally, the *A. tumefaciens* cells were collected and resuspended in induction medium (IM) [37] with 200 μM acetosyringone (AS) to an OD_600_ of 0.4, followed by incubation for about 6 h at 28 °C on a rotatory shaker at 200 rpm to an OD_600_ of 0.6.

**Figure 2:**
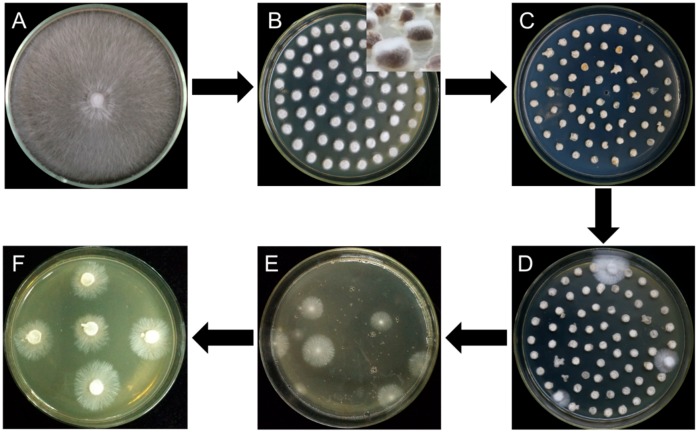
Flow diagram for *Agrobacterium tumefaciens*-mediated transformation of *L. edodes.* (**A**) Strain activation; (**B**) mycelial plugs overgrown with freshly germinated hyphae; (**C**) cocultivation; (**D**) first selection; (**E**) second selection; (**F**) third selection.

All the six different strains, W1, S606, YS55, YS3334, YS3357, and W1-26 were inoculated separately on malt extract, yeast extract and glucose (MYG) agar medium (2% maltose, 2% glucose, 0.1% yeast extract, 0.1% tryptone, 2% agar; commonly used medium for *L. edodes* cultivation) and incubated at 25 °C. In addition, W1 and W1-26 mycelial plugs were grown at 25 °C on the other four media (millet, sawdust medium, 1/2 sawdust medium, and 1/4 sawdust). The millet medium was prepared as previously described [24]. The sawdust medium (improving from sawdust-based cultivation of *L. edodes*) consisted of 7.8% hardwood sawdust, 2% wheat bran, 0.2% gypsum, and 2% agar, while the 1/2 sawdust medium and 1/4 sawdust contained 3.9% and 1.95% sawdust, respectively. When the mycelia covered the medium, all cultures were cut into circular mycelial plugs (Figure 2A), followed by inoculation on a new MYG agar medium and incubation at 25 °C for 2 days (Figure 2B). After the W1 and W1-26 mycelial plugs from MYG medium were inoculated in the millet media, the cultures were gown for 15 days at 25 °C and thoroughly shaken twice daily. 

Mycelial plugs or millet grains overgrown with freshly germinated hyphae were immersed for 20 min in the prepared bacterial suspension of *A. tumefaciens* EHA105 with pCAMBIA-1300-GFP, followed by transferring the samples to the cocultivation medium containing 200 μM AS (Figure 2C). After cocultivation at 25 °C for 2 days, the cultures were rinsed three times with sterile water and then in sterile water mixed with 400 μg·mL^−1^ cefotaxime to eliminate the *A. tumefaciens* cells. The infected mycelia were inoculated to MYG agar medium plates containing moderate hygromycin B and 300 μg·mL^−1^ cefotaxime, then incubated for 14 days at 25 °C (Figure 2D). After the first selection, all transformants on the two plates were ground using a mortar, then mixed with 200 mL 55 °C melting MYG agar medium containing hygromycin B and poured into several plates, followed by incubation for two weeks at 25 °C for the second selection (Figure 2E). Finally, as it is different to incubation all hygromycin-B-resistant colonies germinated in second selection, 30 germinated colonies in each group were picked up and transferred onto fresh MYG agar medium containing hygromycin B for the third selection (Figure 2F). Due to limiting the quantity of hygromycin-B-resistant colonies in third selection, the germinated colonies were counted to evaluate the transformation efficiency. The concentrations of hygromycin B in the selection medium varied with the susceptibility of each strain to hygromycin B are as follows: 4 μg·mL^−1^ for W1, 6 μg·mL^−1^ for S606, 70 μg·mL^−1^ for YS55, 9 μg·mL^−1^ for YS3334, 6 μg·mL^−1^ for YS3357, and 40 μg·mL^−1^ for W1-26. The effect of *A. tumefaciens* strain (EHA105) on transformation efficiency was evaluated by using the W1 MYG mycelial plugs as the recipient material. To investigate whether the inclusion of sawdust can induce the vir genes for T-DNA transfer, the effects of different AS concentrations (200 μM, 100 μM, or 0 μM) on transformation efficiency were evaluated using W1 mycelial plugs cultured in sawdust medium and *A. tumefaciens* AGL1. Each experiment was repeated three times. 

### 2.4. Molecular Analysis of Transformants

After three rounds of selection, plenty of hygromycin-resistant transformants were obtained, and about 20 potential transformants were selected randomly from each group to evaluate the presence of *eGFP* by PCR. Genomic DNA from the transformants and wild types were isolated using the CTAB (hexadecyl trimethyl ammonium bromide) method [38]. The presence of *eGFP* genes was demonstrated by polymerase chain reaction (PCR) analysis using the primer pair GFP-F/GFP-R (Table 1). The PCR amplification procedures started with an initial denaturation at 94 °C for 5 min, followed by 35 cycles of denaturation at 94 °C for 30 s, annealing at 60 °C for 30 s, and elongation at 72 °C for 1 min, with a final elongation at 72 °C for 10 min. To detect the reporter protein eGFP fluorescence, the *L. edodes* transformants were grown on MYG agar medium. When the mycelia grew onto the cover glasses, their fluorescence was detected at 460–500 nm excitation wavelength using a confocal laser scanning microscope (TCS SP8, Leica, Germany). Untransformed colonies of *L. edodes* were used as a negative control.

The positive transformants were cultured through three times transfers in MYG medium without antibiotics. Then the genomic DNA was isolated using Rapid Fungi Genomic DNA Isolation Kit (Sangon, Shanghai, China) according to the manufacturer’s instruction for Southern blot analysis. Briefly, 1 µg of genomic DNA from *L. edodes* transformants or wild type strains was digested with *Hin*dIII. The pCAMBIA1300-g-GFP plasmid linearized with *Hin*dIII was used as a positive control. The digested products were separated on a 0.8% agarose gel and transferred onto a Hybond-N^+^ nylon membrane. A 556 bp *hygR* fragment generated by PCR amplification using primers hpt557-F and hpt557-R (Table 1) was used as a probe to determine the T-DNA copy number per transformant. Probe labeling, hybridization, and signal detection were conducted using DIG High Prime DNA Labeling and Detection Starter Kit I (Roche, Germany) according to the manufacturer’s instructions. The Southern blot analysis was performed by Towin Biotechnology Co., Ltd (Wuhan, China). 

## 3. Results

### 3.1. Effect of A. tumefaciens Strains and AS Concentration on Transformation Efficiency 

**Figure 3:**
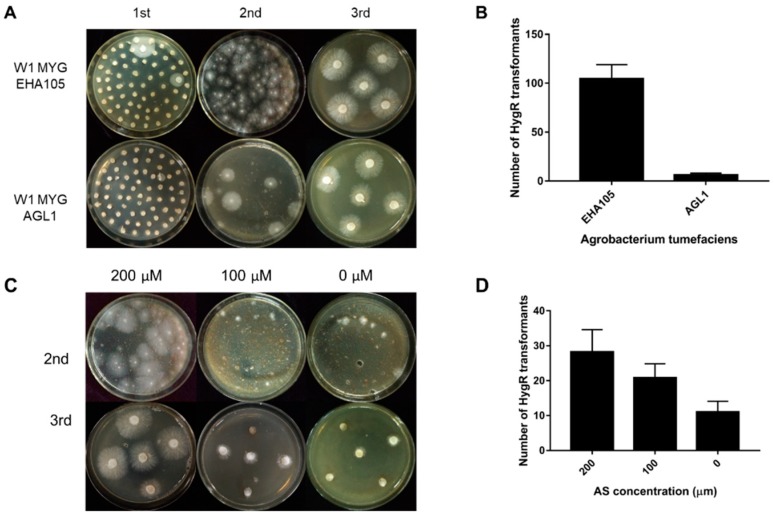
The transformation efficiency as affected by *Agrobacterium tumefaciens* strains and acetosyringone (AS) concentration. (**A**) The hygromycin; (**B**) resistance phenotype of transformation in the 1st, 2nd, and 3rd round of selection when using EHA105 and AGL1, respectively; (**C**) The hygromycin-B-resistant phenotype of transformation in the 2nd and 3rd round of selection when using AS at different concentrations (200 µM, 100 µM, and 0 µM); (**D**) The number of hygromycin-B-resistant colonies per plate in the second selection. MYG: malt extract, yeast extract and glucose (MYG) agar medium.

The ATMT efficiency can be affected by many factors, such as the condition of starting fungal material, *A. tumefaciens* strains and AS concentration. In this study, two different *A. tumefaciens* strains, EHA105 and AGL1 containing the binary vector pCABIAM1300-GFP, were used for transformation of *L. edodes* W1 and evaluation of the transformation efficiency. In the first selection, there were more germinated colonies when EHA105 was used (Figure 3A). In the second selection, when compared with AGL1, EHA105 gained a larger number of hygromycin-resistant colonies per MYG plate for transformation (Figure 3A,B), indicating that *A. tumefaciens* strain EHA105 would help to improve the transformation efficiency. Additionally, in order to know the complex composing in sawdust optimum AS concentration, the induction media and cocultivation media were supplemented with different concentrations of AS (200 mM, 100 mM, and 0 mM). In the second selection, the number of hygromycin-B-resistant colonies per plate increased with increasing AS concentration (Figure 3C,D). After three rounds of selection, we only obtained the hygromycin-resistant transformants in the 200 mM AS treatment. These results demonstrated that the complex contents in sawdust medium could not affect the transformation.

### 3.2. Effect of Activation Medium on the Transformation Efficiency of Dikaryotic and Monokaryotic Strains 

**Figure 4:**
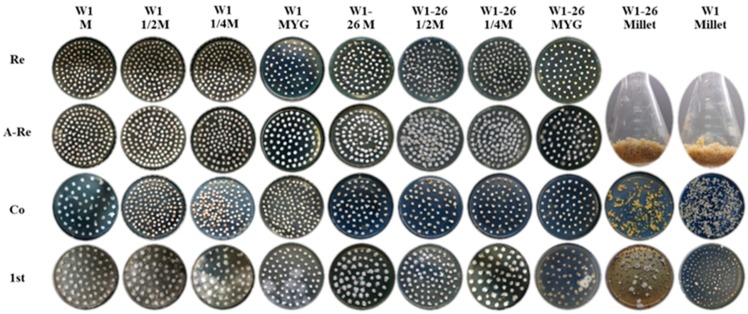
The transformation efficiency as affected by different mediums. Re: Mycelial plugs cultured in MYG medium for rejuvenation; A-Re: Mycelial plugs overgrown with freshly germinated hyphae after rejuvenation in MYG medium; Co: Cocultivation; 1st: First selection. M: Sawdust medium.

To evaluate whether the transformation efficiency will vary with different medium formulations for culturing the recipient mycelium, we chose five different mediums, including millet, MYG medium, sawdust medium (S), 1/2 sawdust medium (1/2 S) and 1/4 sawdust medium (1/4 S). W1 and its monokaryon strain W1-26 were used separately as transformation recipient strains (Figure 4). As showed in Figure 4, mycelium cultured in millet were not incubated in new medium for rejuvenation. The transformation efficiency in each group was assessed by counting the number of hygromycin-resistant colonies and the positive frequency by PCR analysis. The results showed that the 1/4 sawdust medium used for culturing dikaryotic strain W1 could produce the most hygromycin-resistant colonies and showed the highest positive frequency. The more the sawdust was added, the fewer the positive transformants were obtained. When using W1 as the receptor strain, there was a high germination frequency in the first selection, few germinated mycelial colonies in the second selection and low PCR positive frequency of hygromycin-resistant transformants (Table 2). No positive dikaryotic transformant was detected and the fewest hygromycin-B-resistant colonies were obtained in the millet group in the second selection (Table 2). However, when the monokaryotic W1-26 was used as the recipient strain, only the millet group gained hygromycin-resistant transformants, and the positive frequency reached 75% (Table 2). The results indicated that activation medium varies greatly in their effect on the genetic transformation efficiency. Furthermore, dikaryotic and monokaryotic strains also vary obviously in their optimum medium.

### 3.3. ATMT Transformation Efficiency in Different L. edodes Strains

Our previous study has shown that some *L. edodes* varieties are susceptible to *Agrobacterium* infection and easier to gain stable transformants, while some varieties even obtain no stable transformants (in-house data). To broaden the application of the transformation technology in *L. edodes* and to facilitate *L. edodes* research of gene function and genetic improvement, five varieties, W1, S606, YS3357, YS3334, and YS55, were used for a further evaluation of the ATMT transformation efficiency. All strains were grown on the MYG agar medium firstly and successfully transformed using the ATMT method. As the germinated mycelia were ground and plated onto a new selection medium, the number of regerminated mycelial colonies was larger in the second selection, implying a stable transformation frequency. Compared to other strains, the processes involved in T-DNA integration can be assumed to be impaired in W1 due to fewer regerminated hygromycin-resistant colonies and lower positive frequency in the second screening (Table 3). In the YS3334 group, the germination frequency was not the highest in the first selection, while in the second selection, it showed the highest germination and positive frequency of hygromycin-resistant transformants. The YS55 group exhibited the highest germination frequency value of 32.96% in the first selection, while in the second selection, the number of regenerated mycelial colonies and positive frequency were in the middle among all five strains, indicating that YS55 may be superior in T-DNA transfer but inferior in T-DNA integration into genome. The first germination frequency of hygromycin-resistant transformants in S606 and YS3357 was only approximately 1/20 or 1/15 of that in YS55, suggesting the low efficiency of T-DNA transfer to S606 and YS3357 cells. The first selection frequency ranged from 1.41% to 32.96%, the second germination quantity from 7% to 85.25%, and the PCR positive frequency from 2.79% to 85.12%. Considering the various PCR positive frequencies in different strains, the higher positive frequency might indicate the higher efficiency of T-DNA integration into the genomes. Thus, more mitotic stable transformants could be obtained by using YS3334 as the recipient strain.

### 3.4. Molecular Analysis of Transformants 

**Figure 5:**
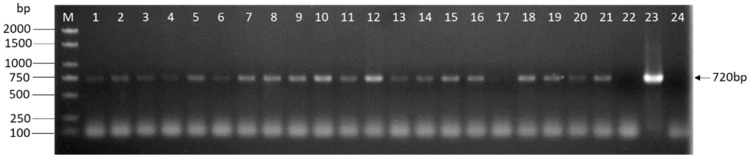
PCR analysis of *L. edodes* transformants. The genomic DNA of the potential transformants was subjected to PCR amplification of *eGFP* contained in the T-DNA regions. The size of the expected PCR product is indicated on the right. Lanes: M: 1kb DNA Ladder (TSINGKE, Beijing, China); 1–21: Different hygromycin-resistant transformants after the third selection; 22: Untransformed W1; 23: Plasmid DNA; 24: ddH_2_O.

After three times of screening, we obtained plenty of hygromycin-resistant transformants. In each group, 21 transformants were randomly selected to evaluate the presence of *eGFP* by PCR. All transformants were used for molecular analysis when the group could not obtain 21 transformants. As shown in Figure 5, bands were detected in some randomly selected transgenic lines or positive control and not in the wild-type strain or ddH_2_O, indicating that the foreign gene *eGFP* was successfully integrated into the genome of *L. edodes*. The result shown in Figure 4 was one assay of Y3334 group.

**Figure 6:**
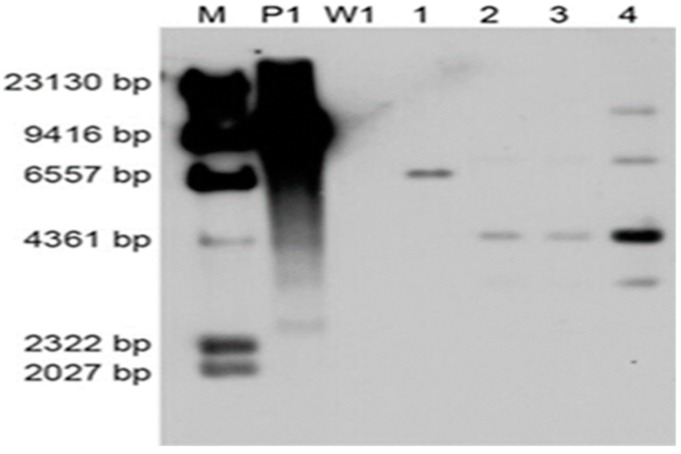
Southern blot analysis of transformants. The numbers (1–4) indicate different transformants using W1 as the recipient strain; M: λ-*Hin*d III molecular maker; P1: Plasmid pCAMBIA-1300-GFP; W1: *L. edodes* strain W1.

To investigate the copy number of foreign DNA integration, four PCR positive transformants were randomly selected for Southern blot analysis using the 640-bp PCR-amplified *hygR* fragment as the probe. Untransformed strain W1 was used as the negative control. All of the four transformants carried the T-DNA insertion, and W1 showed no hybridization bands (Figure 6). The different-size DNA bands on the Southern blot indicated that the T-DNA region was inserted into multiple genomic sites. Moreover, a single copy of the T-DNA was integrated except the fourth transformant.

### 3.5. Screening for eGFP Expression

**Figure 7:**
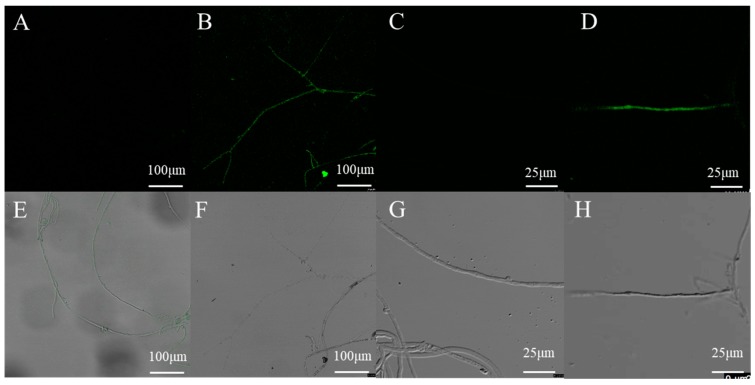
Confocal microscopy analysis of randomly selected transformants harboring *eGFP*. (**B**,**D**,**F**,**H**) the mycelia of one randomly selected transformant harboring *eGFP*; (**A**,**C**,**E**,**G**) the mycelia of untransformed *L. edodes* strain W1.

To verify the expression of the introduced *eGFP* reporter gene, the mycelia of transformants and wild type strain grown without hygromycin B were examined by confocal microscopy. No GFP fluorescence was detected in the wild-type mycelia, and most of the transformants displayed a positive green fluorescent signal (Figure 7). These results indicated that this method is reliable for transformation and the GPD promoter achieves constitutive expression of GFP in *L. edodes*. 

## 4. Discussion

Conceptually, fungal biotechnology is expected to be transformed by the application of genetic transformation technologies. In practice, genetic transformation of fungi meets with many difficulties. Specifically, most of these methods are futile when widely applied to the transformation of basidiomycetes or extensive filamentous fungi [28]. Transformation of mushrooms is very difficult due to the following reasons: Connecting cells with apical growth, thick cell wall, heterokaryosis, and host defense mechanism [22]. Due to its inherent advantages of extensive recipients, steady and random insertion of single copy and high transformation efficiency, ATMT has become a commonly used method for fungal transformation [28]. ATMT was applied to only monokaryon mycelium of *L. edodes* and the transformation efficiency has not been estimated yet [39]. In this work, we applied ATMT to transform six varieties of *L. edodes*, including cultivated strains and wild strains or monokaryon and dikaryon. We achieved the highest germination frequency of 32.96% and the positive frequency of 85.12% in hygromycin-resistant transformants. Previous studies on *A. bisporus* have demonstrated that the culture time of the millet–mycelium complex is critical for high transformation efficiency [24]. The type and age of the tissue being inoculated are known to affect the efficiency of genetic transformation in plants [40]. In the present study, the transformation efficiency was significantly improved by changing the culture medium of recipient mycelia. Fungal cells are connected to form hyphae, which are highly interconnected to form mycelia, making impossible the isolation of single mushroom cells. For this reason, when using mycelia as the recipients, some transformants of filamentous fungi are chimera with the successful transfer of exogenous genes to only part of cells and it is hard to obtain pure and stable transformants. The transformation system described in the present study was added two new steps to optimize the traditional protocol. Firstly, the hygromycin-B-resistant colonies were ground using a mortar or a homogenizer before hygromycin screening to increase the probability of gaining pure transformants. Second, the mycelial plugs were rejuvenated because new and vital tissues are more susceptible to *Agrobacterium* infection [16,22]. Meanwhile, as hygromycin-B-resistant mycelial plugs grown in the first screening were ground, there may be two identical hygromycin-B-resistant colonies that regrow in the second screening, which can be well distinguished from each other by Tail-PCR and Southern blot. 

Bacterial strains and AS have been reported to affect the transformation efficiency [16]. Here, both AGL1 and EHA105 were used as infection bacteria, and EHA105 was found to produce a better result. A previous study has shown that in *Hypsizygus marmoreus*, EHA105 performed better than the other four *Agrobacterium* strains [41], which is consistent with our study in *L. edodes* and another report on *Voluariella volvacea* [42]. Additionally, considering the complex contents in sawdust medium, AS was used in the induction medium and coculture medium at concentrations of 0 µM, 100 µM, and 200 µM, but 200µM AS was found to have the best performance. Previous studies have indicated that the addition of AS during the *Agrobacterium* cocultivation period is essential for transformation, and the induction of the *vir* genes is necessary for T-DNA transfer [43]. As the results of AS screening in this work was similar to archived studies, it is reasonable to assume that the complex contents in sawdust medium could not affect the transformation. The integration of exogenous DNA for the transformation of filamentous fungi is considered to be highly difficult due to the reasons such as connecting cells with apical growth, thick cell wall, heterokaryosis, few molecular biological tools, host defense mechanism, and maintenance of foreign DNA inside mushroom cells [30]. Therefore, the conditions need to be further optimized for improving the transformation efficiency.

Previous reports of ATMT in fungi have indicated that bespoke recipient materials are essential for the efficient transformation of each species. For instance, efficient transformation could be achieved by mycelium for *Suillus grevillei*, protoplasts for *H. marmoreus*, and mycelium pellets for *V. volvacea* [41,42,43]. Bespoke conditions and time-consuming material preparation blocked the establishment of efficient and broadly applicable methods for gene delivery. In the present study, the mycelium was the only recipient and the transformation efficiency was improved by changing the medium used for mycelial incubation. The results suggested that the monokaryon and dikaryon strains from the same variety have their respective specific medium for transformation. During the experiment, the hyphae of monokaryon strains incubated in millet were observed to be healthier, just as described in *A. bisporus* [24]. As different mediums contain diverse nutrients, the morphology and vigor of mycelium varied with medium. Interestingly, the results demonstrated that the less the content of sawdust, the higher the efficiency for gene induction. As we assumed that some compounds in the sawdust could not be detrimental to transformation, a possible explanation is that more sawdust in medium can cause the formation of stocky hyphae and thick cells. Collectively, during *L. edodes* transformation, the medium used for incubating the recipient mycelium showed a notable impact on *Agrobacterium* infection, suggesting an optimal medium for transformation material is essential for enhancing transformation efficiency and transformant stability. 

Furthermore, the genotype of the plant recipient is considered as a crucial factor that can hardly be overcome or complemented through optimizing other external factors [31]. Simmonds et al. performed the genetic transformation of 12 different soybean varieties simultaneously, with the transient transformation frequency of the *GUS* gene ranging from 27 to 92%, and only Accolibri variety gained transgenic plants [44]. To further explore the transformation potential for different *L. edodes* varieties, we applied the transformation system to two cultivated varieties (namely W1 and S606), three wild varieties (namely YS55, YS3334, and YS3357), and one monokaryon strain (W1-26), which achieved efficient transformation frequencies as well (Table 3). Our results showed that while some varieties are more recalcitrant to genetic transformation than others, all the varieties can be infected by this system. So far, few reports have concentrated on the relationship of genotype with genetic transformation in mushrooms, despite extensive reports about that in plants [44,45]. Analyses of the global gene expression patterns across the transformation process in different rice varieties revealed major differences in the expression of genes in response to *Agrobacterium* including genes involved in response to stress or biotic stress, primary and secondary metabolisms, regulation of gene expression, and transport as well as cell proliferation [45]. Considering the great effect of genetic background, the variety differences can be assumed to be largely responsible for the variations in the efficiency of T-DNA integration into the genomes. We also found that the genotype of the recipient mycelium can affect the efficiency of genetic transformation in filamentous fungi, thus choosing a suitable variety for genetic transformation or function analysis is vitally important. 

## 5. Conclusions

In this study, the efficient and simple ATMT protocol was applied successfully not only to both monokaryon and dikaryon strains of *L. edodes*, but also to other four varieties including wild strains and cultivated strains. In order to improve transformation efficiency, the protocol of the *Agrobacterium*-mediated *L. edodes* transformation was optimized in *Agrobacterium tumefaciens* variants and two detail operation procedures. Thus, the optimized genetic transformation method has become more practicable and can be easily implemented in more filamentous fungi by most laboratories. It was firstly reported that the medium composition for recipient incubation could significantly affect the transformation efficiency. This study has paved the way for functional genomic research of this edible mushroom *L. edodes* and other basidiomycetes or filamentous fungi.

## Figures and Tables

**Table 1:** The primers used in this study.

Primer ^a^	Sequence (5′ to 3′)
Pgpd-F	ccacctcaaacttcggaattcGCAGTCAATGGATTGGAGTGTATT
Pgpd-R	gctcaccatCGAAGTTTGAGGTGGTTGCG
gfp-F	ctcaaacttcgATGGTGAGCAAGGGCGAGG
gfp-R	tctagaggatccccgggtaccTTACTTGTACAGCTCGTCCATGCC
EGFP-F	ATGGTGAGCAAGGGCGAGGA
EGFP-R	TTACTTGTACAGCTCGTCCATG
hpt557-F	ACACTACATGGCGTGATTTCAT
hpt557-R	TCCACTATCGGCGAGTACTTCT

^a^ Lowercase bases denote the homologous arms for constructing the vector using ClonExpress Multis One Step Cloning Kit.

**Table 2:** The effect of activation medium and strain karyotype on transformation efficiency of *L. edodes.*

Medium	Strains					
	W1			W1-26		
	1st (%) ^a^	2nd ^b^	PF (%) ^c^	1st (%) ^a^	2nd ^b^	PF (%) ^c^
MYG	12.26 ± 2.04	7.00 ± 4.69	2.79 ± 3.23	16.51 ± 3.77	7.50 ± 2.08	NA
M	100.00 ± 0.00	8.00 ± 5.60	2.72 ± 3.17	50.00 ± 0.00	27.75 ± 3.59	NA
1/2 M	95.00 ± 3.56	18.00 ± 4.32	9.59 ± 8.92	17.69 ± 4.63	28.00 ± 5.10	NA
1/4 M	87.00 ± 2.43	21.00 ± 2.94	30.00 ± 7.07	15.79 ± 3.89	27.75 ± 5.32	NA
Millet	5.14 ± 2.05	1.75 ± 1.50	0.00 ± 0.00	96.22 ± 5.43	58.75 ± 10.78	75.48 ± 4.31

^a^ Mean germination frequency per assay ± SE (standard error) in the first selection. ^b^ Mean number of hygromycin-B-resistant colonies per plate ± SE in the second selection. ^c^ Mean positive frequency (PF) of polymerase chain reaction (PCR) analysis per assay ± SE after the third selection. NA indicates no hygromycin-B-resistant colonies detected after three rounds of selection; M: Sawdust medium.

**Table 3:** *Agrobacterium*-mediated transformation (ATMT) transformation efficiency of different *L. edodes* variants.

Testing Item	Variety				
	W1	S606	YS3357	YS3334	YS55
1st (%) ^a^	12.26 ± 2.04	1.41 ± 0.40	1.97 ± 0.41	7.27 ± 2.94	32.96 ± 10.82
2nd ^b^	7.00 ± 4.69	49.50 ± 4.43	79.00 ± 8.83	85.25 ± 8.54	61.00 ± 16.02
PF (%) ^c^	2.79 ± 3.23	18.86 ± 8.10	26.98 ± 14.55	85.12 ± 14.31	34.47 ± 16.62

^a^ Mean germination frequency per assay ± SE in the first selection. ^b^ Mean number of hygromycin-B-resistant colonies per plate ± SE in the second selection. ^c^ Mean positive frequency of PCR analysis per assay ± SE after the third selection.

## Data Availability

All data generated or analyzed during this study are included in this published article.
